# Comparison of the In Vitro Drug Release Methods for the Selection of Test Conditions to Characterize Solid Lipid Microparticles

**DOI:** 10.3390/pharmaceutics15020511

**Published:** 2023-02-03

**Authors:** Eliza Wolska, Martyna Szymańska

**Affiliations:** 1 Department of Pharmaceutical Technology, Medical University of Gdansk, Hallera 107, 80-416 Gdansk, Poland; 2 Student Chapter of the International Society of Pharmaceutical Engineering (ISPE), Hallera 107, 80-416 Gdansk, Poland

**Keywords:** solid lipid microspheres, solid lipid microparticles, release study, dissolution test, cyclosporine, indomethacin

## Abstract

The release profiles of active substances from microspheres are one of the most important features in solid lipid microparticles (SLM) characterization. Unfortunately, the results of the dissolution tests are largely dependent on the chosen method and test conditions, which in relation to novel dosage forms, such as dispersions of lipid microspheres, are not clearly defined in international compendiums and guidelines. This makes it impossible to compare the results of different studies. The aim of the research was to identify the factors most influencing the variability of the obtained results. An attempt was also made to select the most appropriate method for testing drug substance release from SLM. Various dissolution methods were employed (method I: without a membrane, method II: in a dialysis bag, and method III: in a Side-Bi-Side chamber), and the obtained release profiles of cyclosporine and indomethacin from SLM dispersions were compared. In addition to the effect of membranes, the types of acceptor fluids were also investigated. Significant differences were observed when testing the SLM formulations under various test conditions. The results were significantly influenced by the selected membrane, the acceptor fluid, or the difference in the concentrations of active substance between the donor and acceptor compartments. The burst effect observed in some experimental methods was not noticed in other conditions. At this stage, the method with a dialysis bag has been selected as the most suitable, while the methods without the membrane can only play a complementary role.

## 1. Introduction

Lipid-based drug delivery system is a promising option, particularly due to its biocompatibility. Therefore, solid lipid microparticles (SLM) and solid lipid nanoparticles (SLN) have attracted tremendous interest as drug carriers for poorly water-soluble molecules [1,2,3,4,5,6,7]. SLM dispersions are physicochemical stable, suitable for autoclaving, and seem to be feasible for scale-up [8].

Based on the available studies [4,9,10,11,12], it can be concluded that SLM has proved to be a suitable carrier for local and systemic drug delivery. The use of micron- and nano-sized systems based on lipid carriers allows to improve bioavailability or to mask the taste of the active pharmaceutical ingredients (API) but can also increase the physicochemical stability of API by protecting it against enzymatic or chemical degradation [8,13].

In particular, however, the main advantage of SLM is the prolonged release of API due to the matrix that is solid at body temperature. Moreover, solid, multicompartment lipid preparations offer the possibility of modulating the drug release depending on the degree of API incorporation in the lipid matrix or on its surface [12].

Morphology, particle size, and drug loading are important quality attributes of SLM [14,15]. Each of these features determines the properties of the dosage form and is individually assessed at the development research stage. However, because the drug release behavior is the most critical for in vivo performance, the most important study of a dosage form is the dissolution study, the results of which take into account the simultaneous influence of all the above factors. Similar to other drug formulations, in vitro drug release tests are not only carried out at the SLM development stage. Furthermore, at the manufacturing stage, it is necessary to perform an in vitro release test to evaluate any changes in the production process and thus monitor batch-to-batch variation and ensure quality control [16,17].

Standard dissolution testing, according to United States Pharmacopeia (USP), European Pharmacopeia (EurPh), and Food and Drug Administration (FDA) guidelines, is mostly limited to the classical dosage forms and is inappropriate for multicompartment dispersed systems. Both a paddle apparatus and a flow-through cell can be used for determining drug release from microspheres formulations; however, they are not a perfect solution for testing the microparticulate formulations and require modification, for example, the use of appropriate adaptors [17,18]. Therefore, for non-standard drug carriers, modifications to compendial methods are considered, as well as non-pharmacopoeial methods are developed and applied, taking into account specific characteristics of a tested product. This mainly includes the selection of the dissolution apparatus and the acceptor media. Different experimental setups can influence stirring conditions, sink conditions, and others, thus affecting drug release from the formulation.

In vitro drug release testing methodology used for lipid-based microparticulate systems was divided by Shen and Burgess [18] into three major categories: (i) dialysis techniques, (ii) continuous flow cell methods, and (iii) lipophilic solution floating on the top of the release medium. In practice, the dialysis sac method or the rotating dialysis cell model is most often preferred because the media and the particles are already physically separated by a membrane, and there is no need for extra separation before the sample measurement as well as for the retention of specimens in the system [18,19].

Prolonged release, as well as the effect of burst release, has been reported for lipospheres—both SLN and SLM dispersions [1,20,21,22,23,24,25]. Depending on the route of administration, the burst effect can be useful to obtain a higher initial concentration of API at the application site.

Due to the lack of clear guidelines and the widespread freedom in the selection of apparatus and process conditions, it becomes impossible to compare the research on lipid microspheres conducted by different laboratories. Therefore, it becomes necessary to standardize the method of the research on drug release, for which a comparative assessment of the techniques used so far is needed.

The aim of the research was to use different techniques and conditions to study drug release from SLM and to compare the results in order to indicate the most suitable experimental setups. Cyclosporine (CsA) and indomethacin (Ind) were selected as model APIs. Both of these substances are practically insoluble in water and have been previously studied in the form of SLM. Moreover, CsA is a lipophilic cyclic polypeptide that has already shown promising properties when used in the form of SLM [6,11]. Variability of test conditions included different membranes and acceptor fluids. To the best of our knowledge, these are the first studies of API release from SLM comparing so many different factors and methods, with an indication of the differences resulting from their use.

An attempt was made to indicate the most appropriate testing technique for the evaluation of the release process from a modern, multicompartment drug form, which is the SLM dispersion. Due to the importance of in vitro testing at various stages of drug development and production, it is necessary to develop a commonly accepted research model, which will be considered in international guidelines for testing this dosage form.

## 2. Materials and Methods

### 2.1. Materials

Cyclosporine A (CsA) was purchased from LC Laboratories (Boston, MA, USA), Indomethacin (Ind) from Fagron (Cracow, Poland), Compritol 888 ATO (glyceryl behenate) from Gattefossé (Saint-Priest, France), Tween 80 (polysorbate 80) from Sigma-Aldrich (St. Louis, MO, USA), sodium lauryl sulfate (SLS) and sodium chloride were from POCH (Gliwice, Poland). All other chemicals used were of analytical reagent grade. A Milli-Q system (Millipore, Milford, MA, USA) was employed to obtain high-quality water.

### 2.2. Preparation of SLM Dispersions

Two APIs practically insoluble in water were used in SLM formulations: Ind and CsA (log P 3.4 and 2.9, respectively) [26,27]. The composition of tested formulations is illustrated in Table 1. SLM dispersions were prepared with Compritol as a lipid forming the matrix. The lipid concentration in the dispersions was 10% (*w*/*w*). The API concentration in the dispersion was 2.0% (*w*/*w*) in SLM-CsA and 0.2% in SLM-Ind, which corresponds to a concentration of 20% and 2% relative to the lipid, respectively.

SLMs were prepared using a hot emulsification method [11]. The mixing of the lipid phase with aqueous phase was performed using a high-shear mixer Ultra-Turrax (T25 Janke-Kunkel, IKA Labortechnik, Wasserburg am Bodensee, Germany) after heating both phases to the temperature of 80°C [28]. The resulting emulsion was cooled in an ice bath to solidify lipid microspheres, and the dispersions were stored in a refrigerator.

### 2.3. Characteristics of the Properties of SLM Dispersions

The basic properties of the tested formulations were analyzed as already described in previous reports [11,25,29,30].

The particle size distribution in SLM formulations was measured by laser diffraction with PIDS technology (Polarization Intensity Differential Scattering), (Beckman-Coulter LS 13 320, Indianapolis, IN, USA) using Universal Liquid Module (ULM). The obtained results were presented as: d_10_, d_50_, d_90_, determined as measures of maximum diameter of 10%, 50%, and 90% of the detected particles, respectively [29].

Zeta potential in SLM aqueous dispersions was determined from the electrophoretic mobility using a Zetasizer Nano ZS (Malvern Instruments, Worcestershire, UK) after dilution of SLM in water (1:1000) at 25 °C [11].

The distribution of CsA or Ind between the individual phases of the SLM dispersion was determined as follows [30]. The fraction of free API dissolved in the aqueous phase of the SLM dispersion was determined after ultrafiltration. The sum of the API amount located in the aqueous phase and at the surface of the particles was determined in the supernatant after dilution of SLM formulation with methanol, vortexing, and centrifugation. The amount of the API localized in the interphase was calculated by subtracting from this quantity the quantity found in the aqueous phase (determined after ultrafiltration). The remaining amount was considered as the API fraction incorporated in the lipid matrix [30].

A scanning electron microscope (SEM) Phenom Pro (Phenom World Thermo Fisher, Eindhoven, The Netherlands) was used for morphological evaluation of tested microparticles. SLM dispersion was applied to the carbon adhesive tape fixed to the standard sample holder, water was evaporated from the sample at ambient conditions, and the sample was coated with a thin layer of gold [29]. Acceleration voltage of 10 kV was applied to record images at a magnification of 20,000**×**. The surface visualization and lipid microparticle morphology assessment were performed both before and after the release experiment.

### 2.4. Solubility Studies of Tested APIs in Acceptor Media

The solubility of CsA and Ind was determined in water and in acceptor media used in the release study, including: 0.5% sodium lauryl sulfate solution and 3% or 5% polysorbate 80 solution. In test tubes, an excess of CsA or Ind was mixed with the tested solvents under ambient temperature on a magnetic stirrer with a rotation speed of 300/min. After 24 h, the final suspensions were centrifuged at 3500× *g* rpm for 5 min and filtered through a cellulose acetate membrane filter (Alchem, Torun, Poland) with a pore diameter of 0.2 µm. The concentration of API was determined by high-performance liquid chromatography (HPLC) after appropriate dilution with methanol. The solubility test was carried out in triplicate.

### 2.5. Drug Release Study

**Figure 1:**
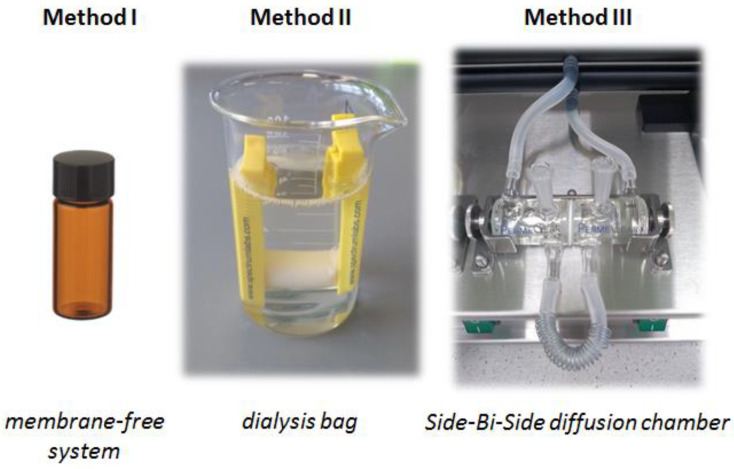
Methods used to test drug substance release from SLM.

The release study was conducted using three methods: Method I—in a membrane-free system—Method II—in a dialysis bag—and Method III—in a Side-Bi-Side diffusion chamber (Figure 1).

The following acceptor media were used: 0.5% sodium lauryl sulfate solution (S) and 5% Tween 80 solution (T), also with the addition of sodium chloride for isotonicity (SN and TN, respectively). In one of the studies (method II), a 0.5% solution of sodium lauryl sulfate with the addition of sodium chloride to hypertonicity (S/Hyper) or ethyl alcohol to isotonicity (S/Iso-Et) was also used. All abbreviations used in describing the release studies are summarized in Table 2.

The samples of acceptor media removed at predetermined time intervals in methods II and III were replaced with an equal quantity of the same fresh acceptor media. The concentration of CsA or Ind dissolved in the sampled acceptor fluids was determined by HPLC after appropriate dilution with methanol. The cumulative API released was expressed as a percentage of the theoretical drug content or as the amount of substance diffusing across the membrane surface (in µg/cm^2^).

**Method I**: membrane-free system. In this method appropriate quantity (100 mg) of SLM dispersion was suspended directly in 5.0 mL of the acceptor media, and the test vial was incubated at 37 °C in a mechanical shaking bath (150 cycles/min) [25]. At the predetermined time intervals, samples were taken, filtered through a 0.2 µm filter (Alchem, Torun, Poland), and diluted with methanol. The 5-point release profile required 5 vials, each with 5 mL of dispersion (one separate vial for each time point: 0.5 h, 1 h, 24 h, 48 h, 72 h).

**Method II**: dialysis bag diffusion technique (Figure 1). Dialysis membrane—Dialysis tubing cellulose membrane (flat width 25 mm) with molecular weight cut-off (MWCO) 14 KDa (Sigma-Aldrich, St. Louis, MO, USA) was prepared prior to the study according to the protocol provided by Sigma. In dialysis bag, 5.0 mL of the following tested formulations were placed: SLM dispersion, SLM dispersion diluted with acceptor fluid in the ratio 1 + 1, 1 + 4, 1 + 49, or API suspension (60 mg/mL CsA or 100 mg/mL Ind). The dialysis bag was placed in a glass beaker containing 70.0 mL acceptor medium and incubated at 37°C in a mechanical shaking bath (150 cycles/min). At the predetermined time points, 2.0 mL of the release medium was withdrawn and replaced by fresh fluid. The diffusion surface area was maintained constant (18 cm^2^) by using membranes with the same length and width for all the tests. The test was also carried out in the so-called inverted system (hereinafter abbreviated as Rev, Table 2), in which the acceptor fluid (5.0 mL) was inside the dialysis bag, and the diluted sample of the SLM dispersion was placed outside in the beaker. In this case, samples for the analysis were taken from the dialysis bag.

**Method III**: Side-Bi-Side diffusion chamber (Figure 1). In this method, Side-Bi-Side glass diffusion cell (PermeGear, Hellertown, PA, USA) with the diffusion membrane installed vertically between the donor chamber (1.0 mL) and receptor chamber (5.0 mL), both with magnetic stirrer and thermostated mantle (37°C), were used. The following four membranes (Table 2) were used: (1) dialysis membrane (RD) (Dialysis tubing cellulose membrane, flat width 77 mm, MWCO 14 KDa, Sigma-Aldrich, St. Louis, MO, USA), (2) Cuprophan membrane (C) (MWCO 10 KDa, Agilent Technologies, Santa Clara, CA, USA), (3) cellulose acetate filter (CA) (Membrane Filters 0.2 μm; Sartorius, Göttingen, Germany) and (4) hydrophobic filter (PTFE) (Membrane Filters 0.2 μm; Sartorius, Göttingen, Germany). The diffusion surface area in this system was 0.66 cm^2^. As in method II, not only SLM dispersions were tested, but also API suspensions (see method II).

### 2.6. Analysis of API Concentration by HPLC

Concentration of CsA and Ind in the tested samples was analyzed by reverse-phase high-performance liquid chromatography (RP-HPLC) using Prominence LC-2030C 3D apparatus (Shimadzu Corporation, Kioto, Japan). The basic conditions for performing HPLC analysis are presented in Table 3.

### 2.7. Statistical Analysis

The obtained data were statistically analyzed using a one-way analysis of variance (Anova). *p*-value < 0.05 were considered to be significantly different.

## 3. Results

### 3.1. Characterization of SLM Dispersions

The average lipid particle size in SLM-CsA and SLM-Ind, as assessed by the LD method, was 2 µm with a majority of particles below 5 µm and with no particles exceeding 15 µm. The detailed parameters of the particle size distribution are shown in Table 4. Zeta potential, as an indicator of particle surface charge, was similar for both formulations.

Table 4 also presents the distribution of tested APIs in different phases of SLM suspension as a percentage of the total drug content [30]. In both tested SLM formulations, less than 1.5% of the drug substance was localized in the aqueous phase, while a large fraction (around 75%) was found on the surface of the lipid particles—in the so-called interphase. The rest of API (23–25%) was identified as incorporated in the lipid core. As can be seen from the obtained results, SLM-CsA and SLM-Ind were characterized by a similar percentage distribution of API in individual dispersion phases despite different API content in the formulations (2.0% CsA and 0.2% Ind).

### 3.2. Solubility Studies

The results of the solubility studies are presented in Table 5. The solubility of CsA and Ind in water was very low (<15 µg/mL), which is confirmed by data from the European Pharmacopoeia (both substances are practically insoluble in water). Among surfactant solutions, 0.5% SLS (S) showed the highest solubilization capacity of CsA and 5% Tween solution (T) of Ind, while the solubility of CsA in T and Ind in S was 10 times lower, respectively. Based on the highest solubility, 5% Tween solution and 0.5% SLS solution were chosen, as the main acceptor media, for further studies.

### 3.3. Drug Release Study—Comparison of Test Methods

**Figure 2:**
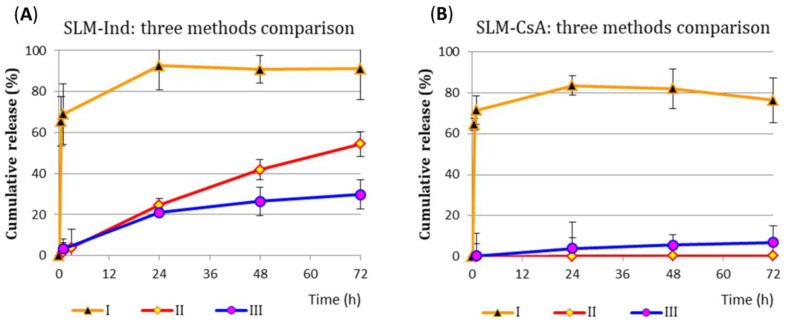
In vitro drug release profiles obtained by three different methods from (**A**) SLM-Ind (into the acceptor fluid with polysorbate) and from (**B**) SLM-CsA (into the acceptor fluid with SLS).

Figure 2 compares the percentages of Ind and CsA release from SLM dispersions obtained by three different methods. The results indicate clear differences between the methods for both APIs.

In a system without a dialysis membrane (method I), a rapid release of a significant fraction of the active substance (50–70%) was observed already within 1 h of the study. The SLM-Ind formulation shows an initial burst release followed by a second slow release phase (extended release) in a time interval depending on the type of acceptor media, while in SLM-CsA, the released substance level reached about 80% of the dose and did not increase at subsequent points time, regardless of the type of acceptor fluid. Some differences in the release of API into the acceptor media with sodium lauryl sulfate or polysorbate were only observed with Ind (data not presented).

Significantly different release profiles were obtained with two other methods of employment dialysis membranes (methods II and III). Primarily, there was no burst effect, and the tested APIs were released much slower (Figure 2A, Ind) or minimally (Figure 2B, CsA). Due to numerous factors that can be modified and have a significant impact on the result of in vitro/in vivo correlation, these two models (methods II and III) were further assessed in subsequent stages of the study.

### 3.4. Drug Release Study—Comparison of SLM with API Suspension

**Figure 3:**
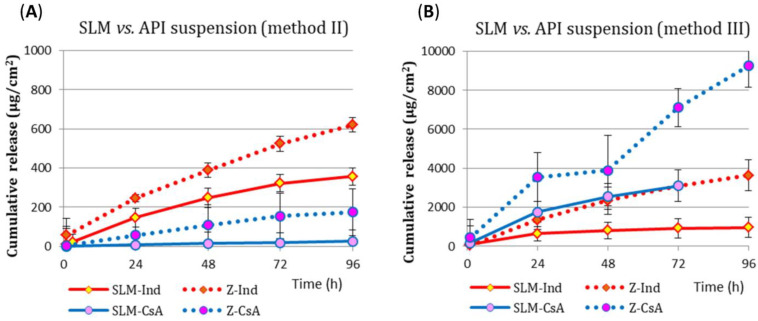
Comparison of Ind and CsA release profiles obtained from SLM dispersion and API suspension (Z) with using: (**A**) method II with RD membrane and (**B**) method III with CA membrane. Ind was released into the acceptor fluid with polysorbate and CsA into the acceptor fluid with SLS.

Figure 3 summarizes the results of the release of both tested substances from SLM and from API suspensions (Z). In each of the analyzed cases, in methods II and III, each drug substance permeated to a greater extent through the diffusion membrane (both RD and CA) from the API suspension (Z) than from the SLM dispersion. In the dialysis bag method (method II), easier permeation of Ind is clearly visible (Figure 3A). In the Side-Bi-Side chamber (method III), the cellulose acetate membrane more easily penetrates CsA (Figure 3B). Furthermore, in this method, the absolute amount of each substance (which permeates per cm^2^ of the CA membrane) is about 10 times greater than in the II method (Figure 3B vs. Figure 3A) with RD membrane.

Regardless of the acceptor fluid type, Ind was released slower if incorporated in SLM (Figure 3A). The drug, both from suspension and from SLM-Ind, was released faster into the isotonic polysorbate solution (TN) than into the other fluids (T, SN, S).

Although several times faster CsA release into the SN media from the CsA suspension was observed than from the SLM-CsA (Figure 3A), the penetration of the drug through the dialysis bag was still at a negligible level in method II (41 µg/cm^2^ from SLM, which corresponds to about 1% of API and 192 µg/cm^2^ from the CsA suspension after 96 h). The observed over time significant slowing down in CsA release into the acceptor media was caused neither by the release of a significant fraction of API from SLM nor by exceeding the solubility or disturbing the sink conditions.

The use of the CA membrane in the horizontal chamber (method III) resulted in a significant difference in the amount of API penetrating into the acceptor compartment from the free drug suspension and from SLM (Figure 3B). In 72 h of the study, CsA release was 7114 µg/cm^2^ from the suspension and 3102 µg/cm^2^ from SLM. The difference in the Ind system was also significant. When a dialysis membrane (RD) was used in method III, similar to method II, the release of CsA from the SLM and the suspension was substantially different, and the differences were much smaller, as described in the following sections.

### 3.5. Drug Release Study—Method II and III

**Figure 4:**
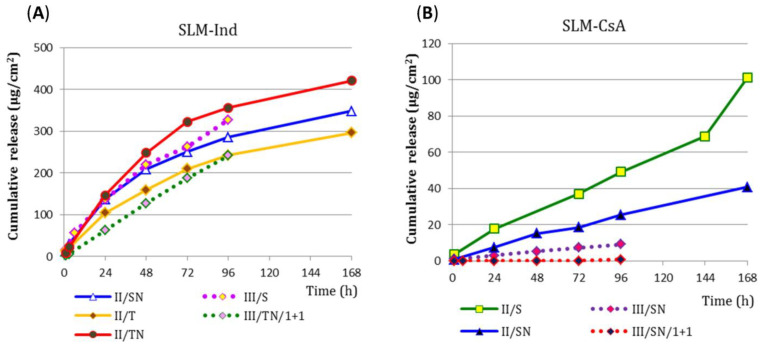
Drug release profiles (*n* = 2–5) from: (**A**) SLM-Ind and (**B**) SLM-CsA to different acceptor fluids obtained by method II or III, through the same membrane (RD, 14 kDa MWCO).

In the next stage, the release from SLM-CsA and SLM-Ind was tested by method II. Using the same system—a dialysis bag made of cellulose membrane with an MWCO of 14 kDa—the CsA release was four times slower than observed for Ind. After 168 h of the study, the cumulative level of CsA released ranged from 40 to 100 µg/cm^2^, while at the same time, from 300 to 400 µg/cm^2^ Ind diffused through the membrane (Figure 4). This effect was observed, although the concentration of Ind in SLM was only 0.2%, while CsA loaded was 2.0% (Table 1). Consequently, 75% of Ind was released from the SLM formulation after 168 h of testing (Figure 4A), while less than 1.5% of CsA was released in any of the SLM-CsA trials (Figure 4B). There were no significant differences in the release of both Ind and CsA depending on the sample volume in the donor compartment (3.0 mL vs. 5.0 mL, not shown in Figure 4). Some differences in the release rate occurred depending on the type of acceptor fluid used in the study (e.g., T vs. TN in Ind or S vs. SN in CsA). Increasing the SN acceptor fluid volume from 70.0 mL to 100.0 mL also had no effect on CsA release, (sink conditions were maintained in both cases).

When the same membrane was used in method III as in method II, no change was observed in the release of Ind from SLM into the sodium lauryl sulfate fluid (Figure 4A). In contrast, the reduction in drug release (expressed in µg/cm^2^) was evident for SLM-CsA (Figure 4B). Diluting the SLM dispersion with the acceptor fluid (1 + 1) in the donor compartment resulted in a reduction in the amount of API released to the acceptor compartment, regardless of the tested substance (Figure 4A,B). A similar effect was observed in the II method after diluting SLM in the dialysis bag (see also Section 3.7).

**Figure 5:**
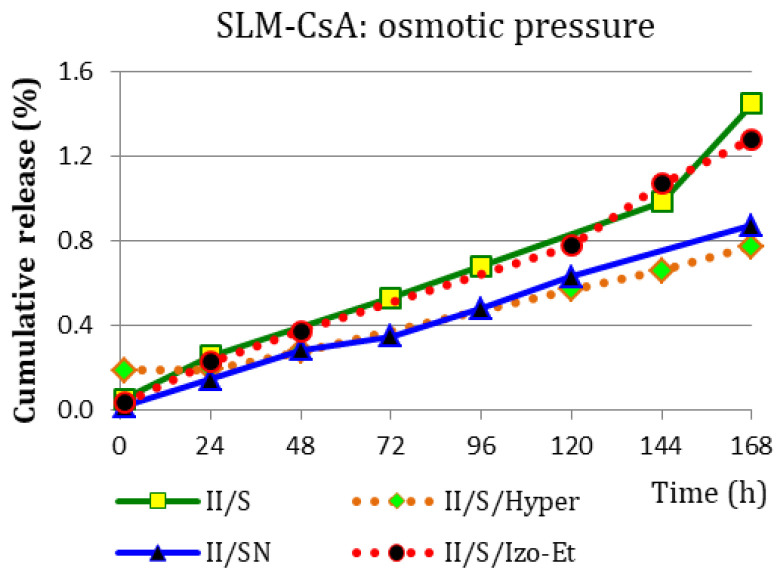
Drug release profiles (*n* = 2–5) obtained with the method II from SLM-CsA in acceptor fluids with different osmotic pressure.

Due to the ambiguous influence of the osmotic pressure of the acceptor media on the CsA penetration through the diffusion membrane (Figure 4), an additional experiment was carried out with a hypertonic acceptor liquid and an ethanol-containing fluid (Table 2). It is worth noting that in the described model of method II (Figure 5), there is practically no CsA release (<1.5% after 168 h of the test, regardless of the acceptor fluid). The observed differences in the amount of CsA released into the hypotonic (S) and isotonic (SN/Izo-Et) acceptor media versus isotonic (SN) and hypertonic (S/Hyper) acceptor fluids increased slightly with the duration of the study (Figure 5).

### 3.6. Drug Release Study—Method III, Membrane Effect

**Figure 6:**
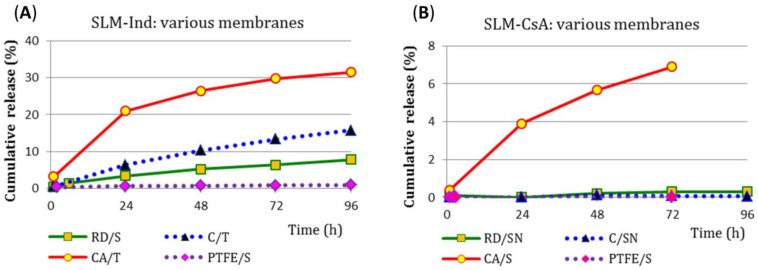
(**A**) Ind and (**B**) CsA release profiles (*n* = 2–5) obtained by the III method (in a horizontal Side-Bi-Side chamber) with the use of different diffusion membranes.

Due to the very limited permeation, especially of CsA through the RD membrane, the next stage of the research was carried out in a horizontal Side-Bi-Side chamber with the use of various semi-permeable membranes (Table 2). The obtained results for both tested substances are presented in Figure 6. It is clear that the type of membrane used is the most important factor determining the results obtained with method III. The Ind release rate was increasing in the following order of the models: with PTFE, with bag membrane (RD), Cuprophan (C) to CA membrane (Figure 6A). The use of a CA membrane with a pore diameter of 0.2 µm allowed the release of 30% of Ind during 96 h of the test. Moreover, the use of the CA membrane allowed for the release of up to 7% CsA in 72 h, while the use of all other types of membranes resulted only in a trace release of the drug (Figure 6B). The observed CsA release rate in the model with CA membrane was significantly greater than in the case of Ind (for example, at the beginning of the test, CsA: 171 µg/cm^2^/h, while Ind: 97 µg/cm^2^/h).

### 3.7. Drug Release Study—Method II, Effect of Dilution and Reverse System

**Figure 7:**
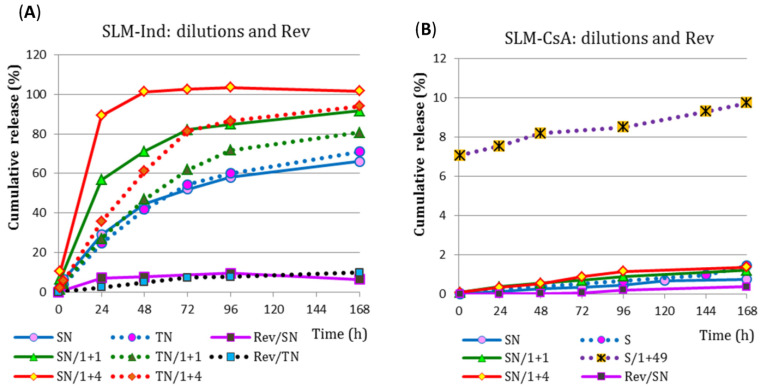
Drug release profiles (*n* = 2–5) from: (**A**) SLM-Ind and (**B**) SLM-CsA, obtained with the II method from SLM undiluted and diluted with acceptor fluid in a dialysis bag, as well as from SLM tested in the standard and reversed (Rev) system.

Comparing the results of the Ind and CsA release from SLM obtained in method II with the dialysis bag, the effect of diluting the SLM dispersion in the bag on the API release was also determined. SLM-Ind and SLM-CsA dispersions were tested without dilution and with a two and five-fold dilution, and additional SLM-CsA was also diluted 50-fold. The obtained results are presented in Figure 7 as the percentage of the total dose.

From the most diluted SLM-Ind (1 + 4), the release of 100% API took place within 48 h, while from the remaining SLM-Ind formulations during 168 h of the study, 70% to 94% of Ind was released. On the other hand, in both acceptor media, the release rate (expressed in µg/cm^2^/h) decreased with the dilution of the SLM dispersion. During the first 48 h of the test, the release occurred faster into the SN media. After this time, this trend was reversed, and the observed differences were smaller than in the first step of the study.

When interpreting the CsA release profiles (Figure 7B), it can be seen that the drug is released and diffusing the membrane to a small extent (less than 100 µg/cm^2^ after 6 days of the test), and this is much slower than in the case of Ind. All observed CsA release profiles are very similar, and the percentage of CsA released (Figure 7B) exceeds 10% only in the formulation diluted before testing (1 + 49), which is only due to the low dose of CsA in the bag. In the remaining systems, after 6 days, the released amount of CsA is below 2%.

The release of API from SLM was also assessed in the dialysis bag method, but in a reversed system (Rev), in which the dialysis bag contained 5.0 mL of isosmotic acceptor fluid was immersed in the SLM dispersion diluted with the acceptor fluid in a beaker. A comparison of these two models is shown in Figure 7. The release of Ind in the reversed system (release into the bag) after 24 h of the test resulted in an Ind concentration of 20 and 57 µg/mL in TN and SN fluid, respectively. This is significantly below the solubility of Ind in these solutions. The obtained concentration of CsA in the SN fluid (2 µg/mL) was even lower. A slight increase in the release rate into the bag and, consequently, an increase in the concentration of CsA in the acceptor fluid was observed after 72 h of the test (Figure 7B), but it was still much below the solubility of API. Although the sink conditions were not maintained in the reversed system, the concentrations determined in the tests suggest that this was not the cause of the observed differences compared to the profiles in the classic system.

### 3.8. Microscopic Evaluation of Microspheres

**Figure 8:**
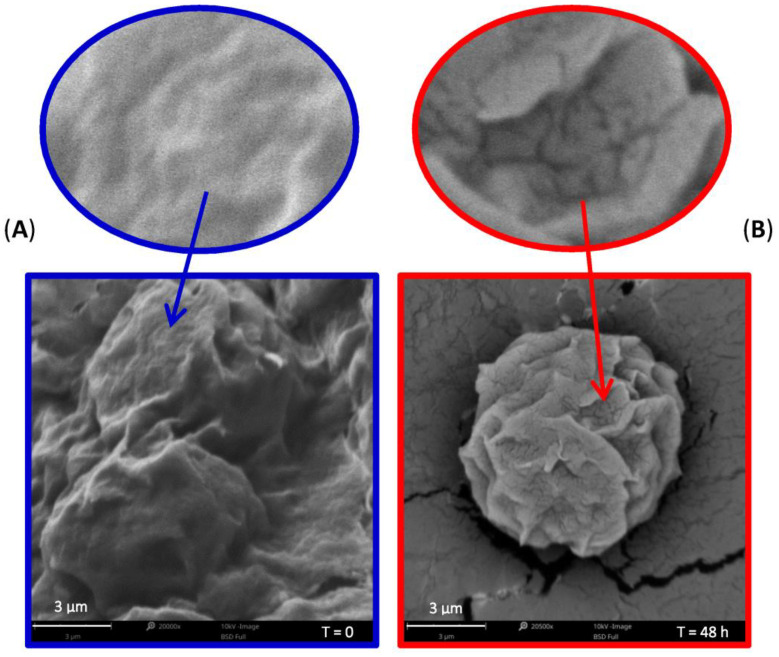
SEM micrographs of the microparticles: (**A**) before and (**B**) after 48 h of release study.

Microscopic evaluation of the investigated microspheres was performed during the release studies to evaluate the changes that had occurred in the lipid matrix due to API release. Lipid microspheres were characterized after preparation and after 48 h of the release study using SEM (Figure 8).

The particles in SLM were spherical in shape with a bumpy, uneven surface, similar for both CsA or Ind formulations. As can be seen in Figure 8, after 48 h of the release test, the shape and size of the CsA-loaded lipid particles remain intact. At the same time, quite distinct defects in the form of cracks and fissures are visible in the structure of SLM-CsA. Similar observations were made for SLM-Ind.

## 4. Discussion

The most important parameter in solid lipid particle characterization is the release rate of the active substance. As already described in previous studies [25], even accurate analyzes of the active substance distribution in the system do not provide such detailed information on the behavior of the API in the formulation as the release study, which also includes the influence of other factors such as particle size and morphology. Since one of the main aims of microparticle carriers is the prolonged release of the incorporated drug, in vitro release studies have to be carried out [14]. At the same time, due to the innovative nature of the studied dosage form, there are no clear guidelines or a pharmacopoeial method suitable and dedicated typically for the multicompartment lipid system. The published data describe various techniques, apparatuses, and conditions used to test the release of API, which makes it impossible to compare the obtained results, and thus slows down the development of this promising drug carrier.

Therefore, studies were undertaken to compare the release profiles of the two model APIs from SLM, obtained by different techniques in different apparatuses, using different conditions. The more commonly used method with a dialysis membrane was used in two models, as well as a method without a membrane but with a small volume of acceptor fluid. Efforts were made to select the factors that have the greatest impact on the obtained results. An attempt has also been made to indicate the most suitable technique for testing the release in such dosage forms.

### 4.1. Solubility Studies

The solubility of API in acceptor media is one of the most important factors allowing the determination of the sink condition during the release study; therefore, it was assessed in a separate study (Table 5). The saturation solubility of tested APIs was assessed in different solvents to select the best one as acceptor media for release studies. Finally, solutions of both surfactants were used as the release media in the research (sodium lauryl sulfate solution 0.5% and polysorbate solution 5%) in order to verify if the API release was affected by the different solubility of the API in the release media, despite maintaining the sink conditions in each case.

### 4.2. Drug Release Study in a Membrane-Free System (Method I)

In the presented study, SLM formulations with one emulsifier, namely polysorbate 80, were produced. Moreover, the SLM formulations with Ind and CsA (Table 1) were characterized by a similar distribution of API (Table 4). In earlier studies, SLM-CsA were also tested with a lower Tween concentration [25,31,32] and greater drug incorporation in the lipid matrix [11,31], but this time they were not used to eliminate this element of variability in the currently tested systems.

The amount of Ind released from SLM-Ind approached about 100% between 24 and 48 h, while the amount of CsA released after reaching a level of about 80% did not change until the end of the study (Figure 2). A similar effect was achieved in our previous studies [25] in which a significant amount of the API was released into the acceptor fluid in a short time (the so-called “burst effect”). After the rapid release phase, the API was released much more slowly in the next phase. Similar profiles of active substances released using the membrane-free method were also obtained by Pilanya et al. [10] or by other researchers using the paddle method [33] or the dialysis bag method as well [34].

The lipid microspheres were also observed by SEM during the dissolution test. Taking into account factors such as: the amount of API released within 48 h, the lack of factors in the acceptor media affecting the enzymatic degradation of the lipid matrix, and the surface localization of a significant fraction of API in the microspheres (findings based on distribution studies and other research [25]), it is supposed that the observed changes are due to the release of drug substances from the dosage form.

When examining the release in the membrane-free model (method I), no differences were observed in the release profiles into fluids without (S, T) and with sodium chloride (SN, TN). Between liquids with sodium lauryl sulfate (S, SN) and polysorbate (T, TN), only indomethacin showed some differences, which can be explained by the different solubility of Ind (Table 5).

From a practical point of view, an initial burst release may be considered beneficial in terms of achieving the therapeutic concentration of API at the site of drug application in minimal time, followed by sustained release of the drug substance. This effect is absolutely related to the distribution of the API in the SLM dispersion. One of the key factors affecting the release kinetics of API from SLM particles is the way the substance is distributed in SLM dispersion and especially in the lipid matrix [35,36]. As shown by the distribution studies (Table 4), the vast majority (approx. 75%) of API is on the surface of lipid microspheres (in the so-called “interphase”), and only a small amount (0.6–1.5%) of the drug substance is present in the aqueous phase. The remaining API fraction is incorporated in the SLM lipid matrix (Table 4). The amount of API released in the first step (burst effect) is comparable with the amount of API determined at the interphase.

It is obvious that the fraction of API localized on the surface of the lipospheres will be released faster than the drug substance molecules incorporated in the lipid matrix. According to the solid nature of SLM, and thus less drug particle mobility, a prolonged release profile in tested formulations is expected to be achieved [37]. The acceptor fluids used in the research were only water solutions of surfactants, so they did not result in the degradation of the microsphere’s matrix. This explains the behavior of SLM-CsA (release retention at about 80%). At the same time, this mechanism questions the ability of Ind to be incorporated into the lipid matrix to the same extent as CsA.

The release study in a membrane-free model was used for both SLM and SLN studies [30,38,39], although it is not clear whether the observed burst effect is a reflection of the readily available surface-localized API or merely the result of direct dilution and mixing of the lipospheres with a large amount of acceptor fluid.

### 4.3. Drug Release Study in a Membrane Model—Methods II and III

In the next stage, the release study was carried out with the use of semi-permeable membranes in a diffusion apparatus (method III) and in a dialysis bag (method II). The membrane release test is fundamentally different from the non-membrane test since the permeation of API into the acceptor media is limited not only by release from the formulation and dissolution in the acceptor fluid but also by diffusion of the substance through the semi-permeable membrane. The applied membrane retains the SLM in the donor compartment but allows the transfer of the dissolved/released drug molecules into the release media. Moreover, both methods (II and III) differ significantly in the area of the membrane through which the penetration of API into the acceptor fluid takes place, as well as in the volume of the test sample (SLM dispersion) in the donor compartment. Therefore, the obtained results are also presented in the form of the amount of released substance per cm^2^ of the membrane at a given time point (µg/cm^2^) in order to standardize the presented results and facilitate comparison (Figure 3).

The fundamental difference between the membrane model and the membrane-free model was observed. In studies with the membrane, no effect of rapid release of a significant fraction of the drug (burst effect) was observed, as in the case of the model without the dialysis membrane. Moreover, in the models with membranes, significant differences were visible between the two tested substances, while the release profiles were very similar in the method without the membrane. These effects confirm the essential influence of the membranes and models.

Experiments with a pure substance in the form of an Ind or CsA suspension were also carried out (Figure 3). The release of API from the suspension was slow but faster than from the SLM. Regardless of the test method (II or III), the permeation of both Ind and CsA from the free API suspension was found to be much greater than from the SLM dispersion. In method II, the rate of diffusion of both drug substances through the same membrane depended on the type of acceptor fluid, which was also observed in other experiments. In the III method, the amount of API diffusing between the compartments was strictly dependent on the type of selectively permeable membrane separating these compartments. There was a huge difference in the amount of drug released from SLM and suspension of API (for both Ind and CsA) when the barrier was a CA membrane. A much smaller difference was observed when using the RD or C membrane, mainly due to the further described lower ability of tested substances to penetrate these membranes.

The main factor of the membrane deciding the course of the process is the so-called molecular weight cut-off (MWCO). In accordance with the manufacturer’s declaration of the membrane, MWCO designates a molecular weight below which molecules can move freely through the membrane [40]. There are no guidelines for the selection of that parameter of the membrane according to the molecular weight of the test substance. There are some hints in the scientific reports based more on the researchers’ own experiences. For example, Xu et al. [41] recommend that the cut-off point should be about 100 times the molecular weight of the API. However, based on the analysis of literature data, it can be concluded that the commonly used MWCO is 12–70 times greater than the molecular weight of the tested substance [37,41,42,43]. The molecular weight of CsA and Ind compared to the MWCO of the dialysis membrane used in our studies (14 kDa) results in a 12 times and 40 times greater cut-off point relative to CsA and Ind, respectively. On this basis, one would expect the free movement of the tested APIs through the diffusion membrane.

Due to the movement of the membrane with the seals against the donor and acceptor chambers (III method), there was a risk of reducing the already small permeation area. It was observed that the tightness of the system and the ease of its preparation (proper mounting of the membrane) for the test, among other things, depended on the type of membrane used. Moreover, after the test, heterogeneity of dispersion was noticed in the donor compartment, where the lipid microspheres accumulated to a large extent from the membrane side (where the chamber is structurally constricted), despite the use of magnetic stirring in both parts of the chamber throughout the test. This phenomenon was not observed when the SLM dispersion in the donor compartment was diluted with the acceptor media prior to testing.

Further experiments were conducted using different membranes in model III. The pore size of the membrane through which API permeates, as well as its hydrophilic/hydrophobic properties, are also crucial for the penetration of API into the acceptor compartment and, consequently, for the observed release profiles. The application of the CA membrane as a selective partition between the compartments resulted in a two-fold (Ind), and in the case of CsA, even a several dozen-fold increase in API penetration into the receptor media (Figure 6). For the PTFE membrane, although it had a pore size the same as CA (0.2 µm), the permeation was the lowest of all systems tested (<1% Ind) or undetectable (CsA). This leads to the conclusion that better diffusion of API is provided by a hydrophilic membrane (CA with 0.2 µm pore size).

The comparison of the release profiles observed for SLM and pure substances proves that the penetration of both APIs is not limited only by the capacity of diffusion through the membrane but also by the SLM carrier, which is a prolonged release system (Figure 3).

### 4.4. The Effect of Acceptor Fluid in the Membrane Model

Research on free API and SLM confirms that both tested drug substances diffuse through the semi-permeable membranes to a different extent (Figure 3A vs. Figure 3B). The influence of the solubility of API in the solution used as acceptor media or the difference in concentration on both sides of the membrane, which is the driving force of the process, are significant factors that cannot be ignored either.

In models with a membrane, the osmotic pressure on both sides of the membrane is also of particular importance. Therefore, in the acceptor fluid, apart from the surfactant used due to the limited solubility of the tested APIs, there should also be excipients that ensure adequate osmotic pressure. In the donor compartment, this pressure should be equal to or greater than the pressure in the acceptor compartment, which prevents water from flowing in a direction consistent with the pressure differential but opposite the direction of API diffusion/release. Such flow may disturb and slow down API penetration into the acceptor compartment.

In method II, a slightly lower release of Ind into the SN fluid can be noticed compared to the TN (Figure 4A), which is due to the solubility of the API (Table 5). At the same time, a difference in the release of Ind to the T fluid with and without the addition of sodium chloride was observed (Figure 4A). The described phenomenon is confirmed by the swelling of the dialysis bag. This effect does not occur when the dialysis bag is placed in an isosmotic acceptor fluid (TN). Therefore, an acceptor fluid frequently used is PBS [43,44], although the use of pure water is not rare [45,46].

The results obtained in the release studies of CsA from SLM and isosmotic acceptor fluid (Figure 4B) are different from those described for Ind. Although the differences were not significant, the systems without sodium chloride (S and S/Izo-Et with ethanol) as the acceptor media were characterized by faster release (Figure 5) in comparison to SN and S/Hyper, contrary to the thesis about hindered API transfer. The obtained results confirm the behavior of CsA different from Ind in the tested model. Due to the high volatility of ethanol and the risk of changing the concentration, osmotic pressure, and composition of the acceptor fluid during the test, the use of this acceptor medium was abandoned.

SLM dispersions were also examined after dilution in the donor compartment with the same acceptor media into which the release took place, which mimics the conditions of method I in which the SLM are directly diluted with an acceptor fluid. It can be concluded that the greater the dilution, the greater the percentage (if expressed in the percentage of the total dose) of the Ind dose released through the membrane (Figure 7A). However, if the profiles are expressed in the units µg/cm^2^, the smaller the dilution was, due to the concentration gradient effect, the permeation rate was higher (a smaller difference in concentration on both sides of the membrane in diluted systems results in a slower release to the acceptor media). Moreover, in diluted systems, a faster release of Ind into the SN media than into the TN was observed (which is in contradiction with the previously observed effect related to the 10-fold better solubility of Ind in the polysorbate solution than in sodium lauryl sulfate solution). The slowdown release in TN fluid in the semi-permeable membrane model may be due to the formation of micelles inside the bag, which solubilizing and retaining the API in the initial phase slows down the diffusion of Ind through the membrane into the acceptor media. At a later stage, the release to TN is faster than to SN fluid because the effect of better solubility begins to dominate. As can be seen from the example presented, the use of the research model with a diffusion membrane significantly influences the obtained results and the observed phenomena.

Comparing the formulations (SN/1 + 4) with Ind and (S/1 + 49) with CsA (Figure 7A,B), it can be concluded that despite the fact that both dialysis bags contain the same amount of API (about 2 mg), the percentage of released the dose is 100%/48 h of Ind and 10%/168 h of CsA, which clearly confirms the much slower and more difficult penetration of CsA through the dialysis membrane in method II.

Finally, the release of API from SLM diluted in acceptor media in the so-called reverse system (Rev) was also tested. This system provided a different and better mixing of the donor compartment located in the beaker. However, this did not have a positive effect on the rate of API permeation through the diffusion membrane. The concentration of the dispersion in the donor compartment turned out to be a much more important factor influencing the permeation through the membrane. The greater the concentration difference on both sides of the membrane (classical system with SLM in a bag immersed in the acceptor media), the faster the penetration of API through the membrane was observed. When the SLM dispersion was diluted with a large volume of acceptor fluid in the beaker (Rev), the penetration of API into the dialysis bag with acceptor media was much slower (Figure 7). The same situation with a decrease in permeation rate occurred in the example described above when the SLM dispersion was diluted with acceptor fluid in the dialysis bag (Figure 7).

## 5. Conclusions

The conducted research clearly shows that the applied dissolution test method is crucial for the obtained results. The use of different methods leads to different release profiles. Depending on the method, the burst effect or its absence was observed. The results were significantly influenced by the selected semi-permeable membrane (due to MWCO), the acceptor fluid, osmotic pressure, or the difference in API concentrations between the donor and acceptor compartments.

In the methods using semi-permeable membranes, the selection of a membrane with appropriate properties is fundamental. The key advantages of method II include the separation of SLM from the acceptor fluid, no need to filter the collected sample as well as the retention of the specimen in the system. The dialysis bag capacity and the acceptor fluid volume can be adjusted to the sink conditions so that the conditions used allow the release of the entire dose of API from SLM.

The use of method III in SLM release studies generally resulted in observations similar to method II. The key difference in method III was observed only when a different than RD semi-permeable membrane was used. Moreover, method III (the Side-Bi-Side chamber) turned out to be the most demanding from an analytical and technical point of view due to the relatively small permeation surface area and episodes of the leakage of release media from the acceptor chamber.

The phenomenon of the slower release of API from the SLM dispersion compared to the API suspension (free drug) confirms the influence of the dosage form on the release rate and the slow-release effect provided by the solid lipid matrix of the microspheres.

Taking into account all the above differences, it is extremely important that the dissolution tests are always carried out with a strictly defined method under the same conditions. In order to enable the efficient development of modern dosage forms, such as SLM, it is important that regulatory authorities indicate and recommend the method and basic guidelines for assessing the quality of the formulation in the form of SLM, allowing the results to be compared without the risk of obtaining erroneous conclusions for methodological reasons. Therefore, the described research was undertaken in order to facilitate the identification of the method that will be the most suitable for testing the release of API from SLM and, as a rule, used by everyone. 

In our opinion, at the present stage of research, method II with a dialysis bag should be considered the most appropriate, while methods based on the direct mixing of SLM with an acceptor media (the I method) may only play an auxiliary role in confirming or verifying the obtained results, mainly due to the correlation of the amount of API released in the burst effect with the amount of drug substance determined at the interphase.

## Figures and Tables

**Table 1:** The composition of the investigated SLM formulations.

Formulation	Content (% *w*/*w*)				
	CsA	Ind	Compritol	Tween 80	Water
SLM-CsA	2.0	-	10.0	5.0	83.0
SLM-Ind	-	0.2	10.0	3.0	86.8

**Table 2:** The abbreviation system introduced in the release study.

Abbreviation	**Method**
I	membrane-free system
II	dialysis bag diffusion technique
III	Side-Bi-Side diffusion chamber
Abbreviation	**Acceptor media**
S	0.5% sodium lauryl sulfate solution
SN	0.5% sodium lauryl sulfate solution with sodium chloride to isotonicity
T	5% Tween 80 solution
TN	5% Tween 80 solution with sodium chloride to isotonicity
S/Hyper	0.5% solution of sodium lauryl sulfate with sodium chloride to hypertonicity
S/Izo-Et	0.5% solution of sodium lauryl sulfate with ethyl alcohol to isotonicity
Abbreviation	**Membranes used in method III**
RD	cellulose membrane with molecular weight cut-off 14 KDa
C	Cuprophan membrane with molecular weight cut-off 10 Kda
CA	cellulose acetate membrane filters with 0.2 μm pores
PTFE	hydrophobic membrane filters with 0.2 μm pores
Abbreviation	**Others**
Z	API suspension (suspension without SLM)
Rev	test conducted using method II, but in an inverted system

**Table 3:** HPLC analysis parameters for individual active substances.

HPLC Parameters	CsA	Ind
Column	LiChrospher 100 RP-18, 250-4	LiChrosorb RP-18, 125-4
Mobile phase	acetonitrile/water/t-butyl methyl ether/ortophosphoric acid (520:430:50:1 *v/v*)	acetate buffer/methanol (35:65 *w*/*w*)
Temperature	80 °C	30 °C
Flow rate	2 mL/min	1 mL/min
Wavelength	210 nm	215 nm

**Table 4:** Characteristics of tested SLM dispersions (mean ± SD, *n* = 3).

Formulation	Particle Size (µm)			Zeta Potential (mV)	API Distribution (%)		
	d_0.1_	d_0.5_	d_0.9_		Aqueous Phase	Interphase	Lipid Matrix
SLM-CsA	0.77	1.40	2.62	−22 ± 0.6	0.6 ± 41	74.8 ± 2.6	24.6
SLM-Ind	0.64	1.78	5.04	−27 ± 0.5	1.5 ± 23	75.8 ± 7.7	22.7

**Table 5:** The solubility (expressed in mg/mL) of active substances in water and acceptor media.

Active Substance	Tested Solvents (pH 6.1–7.4)			
	Water	3% Tween 80 Solution	5% Tween 80 Solution	0.5% SLS Solution
CsA	0.0125	0.47	0.66	5.86
Ind	0.0142	0.61	1.24	0.12

## Data Availability

Not applicable.
